# Preoperative Concurrent Chemoradiotherapy Versus Neoadjuvant Chemotherapy for Locally Advanced Gastric Cancer: Phase II Randomized Study

**DOI:** 10.3389/fonc.2022.870741

**Published:** 2022-04-29

**Authors:** Xin Wang, Dong-Bing Zhao, Lin Yang, Yihebali Chi, Hong Zhao, Li-Ming Jiang, Jun Jiang, Yuan Tang, Ning Li, Wen-Yang Liu, Li-Zhou Dou, Shuang-Mei Zou, Li-Yan Xue, Jian-Song Ren, Yan-Tao Tian, Xu Che, Chun-Guang Guo, Xiao-Feng Bai, Yue-Min Sun, Shu-Lian Wang, Yong-Wen Song, Yue-Ping Liu, Hui Fang, Ye-Xiong Li, Jing Jin

**Affiliations:** ^1^Department of Radiation Oncology, National Cancer Center/National Clinical Research Center for Cancer/Cancer Hospital, Chinese Academy of Medical Sciences and Peking Union Medical College, Beijing, China; ^2^Department of Pancrea-gastric Surgery, National Cancer Center/National Clinical Research Center for Cancer/Cancer Hospital, Chinese Academy of Medical Sciences and Peking Union Medical College, Beijing, China; ^3^Department of Medical Oncology, National Cancer Center/National Clinical Research Center for Cancer/Cancer Hospital, Chinese Academy of Medical Sciences and Peking Union Medical College, Beijing, China; ^4^Department of Radiology, National Cancer Center/National Clinical Research Center for Cancer/Cancer Hospital, Chinese Academy of Medical Sciences and Peking Union Medical College, Beijing, China; ^5^Department of Radiation Oncology, National Cancer Center/National Clinical Research Center for Cancer/Cancer Hospital & Shenzhen Hospital, Chinese Academy of Medical Sciences and Peking Union Medical College, Shenzhen, China; ^6^Department of Endoscopy, National Cancer Center/National Clinical Research Center for Cancer/Cancer Hospital, Chinese Academy of Medical Sciences and Peking Union Medical College, Beijing, China; ^7^Department of Pathology, National Cancer Center/National Clinical Research Center for Cancer/Cancer Hospital, Chinese Academy of Medical Sciences and Peking Union Medical College, Beijing, China; ^8^Office of Cancer Screening, National Cancer Center/National Clinical Research Center for Cancer/Cancer Hospital, Chinese Academy of Medical Sciences and Peking Union Medical College, Beijing, China; ^9^Department of Pancrea-gastric Surgery, National Cancer Center/National Clinical Research Center for Cancer/Cancer Hospital & Shenzhen Hospital, Chinese Academy of Medical Sciences and Peking Union Medical College, Shenzhen, China

**Keywords:** neoadjuvant therapy, chemotherapy, chemoradiotherapy, gastric neoplasm, surgery

## Abstract

**Objective:**

We evaluated and compared the efficacy and safety of neoadjuvant chemoradiotherapy (NACRT) versus neoadjuvant chemotherapy (NACT) for locally advanced gastric cancer (LAGC) in a single-center randomized phase II trial.

**Methods:**

Patients with LAGC were enrolled and received either NACT or NACRT, followed by gastrectomy and adjuvant chemotherapy. The primary endpoint was an R0 resection rate.

**Results:**

We enrolled 75 patients: 75.7% (NACT, 28/37 patients) and 76.3% (NACRT, 29/38 patients) underwent surgery; R0 resection rates were 73.0% (27/37) and 73.7% (28/38), respectively. The NACRT group had significantly better major pathological response than the NACT group (37.9% vs 17.9%, p = 0.019). Between-group postoperative complications were not significantly different. The median follow-up was 59.6 months; 5-year overall survival (OS) rate was 50.1% (NACT) and 61.9% (NACRT); neither group reached the median OS; median progression-free survival was 37.3 and 63.4 months, respectively.

**Conclusions:**

S-1-based NACRT did not improve the R0 resection rate, although it presented better tumor regression with similar safety to NACT.

**Trial registration:**

ClinicalTrial.gov NCT02301481

## Introduction

Gastric cancer (GC) is the fifth most common cancer and the third leading cause of cancer-related mortality worldwide (1), seriously endangering human lives and health. Due to the atypical symptoms and lack of screening systems, more than 50% of patients with GC are diagnosed with locally advanced disease at diagnosis (2). Radical resection is the main treatment for locally advanced GC (LAGC), while the clinical benefit of gastrectomy alone or plus adjuvant treatment is extremely limited, with long-term survival rates of <30% (3). Further, the prognosis of LAGC without R0 resection or unresectable LAGC is even worse. Therefore, explorations of neoadjuvant treatment for patients with LAGC have attracted much attention.

In 2006, the landmark MAGIC trial (4) demonstrated that perioperative chemotherapy (ECF: epirubicin, cisplatin, 5-fluorouracil) followed by surgery significantly improved the progression-free survival (PFS) of stage II or higher GC, which other relevant research subsequently confirmed (5). The CROSS study (6) verified that neoadjuvant chemoradiotherapy (NACRT) followed by surgery versus surgery alone for locally advanced esophageal cancer and esophagogastric junction cancer (EGJC) conferred survival benefits. Accordingly, the National Comprehensive Cancer Network guideline recommends both neoadjuvant chemotherapy (NACT) and NACRT as the standard of care for LAGC and locally advanced EGJC. However, no prospective studies to date have directly compared the NACT and NACRT regimens in patients with LAGC.

Therefore, this trial, involving patients diagnosed with LAGC, was aimed at optimizing preoperative therapy by comparing the efficacy and safety of NACRT and NACT.

## Materials and Methods

### Study Design

This was a prospective, parallel, open-label, randomized phase II clinical trial conducted from January 2014 to October 2017. Approval was obtained from the National Cancer Center ethics committee. Randomization was performed by computer-generated allocation, with stratification by clinical T stage (T4 vs. non-T4). All included patients provided written informed consent. This trial has been registered and released with ClinicalTrials.gov (identifier NCT02301481) and followed the Consolidated Standards of Reporting Trials (CONSORT) reporting guideline.

### Eligibility

Patients with pathologically confirmed gastric adenocarcinoma staged as T3–4bN0M0 or any TN+M0 (American Joint Committee on Cancer [AJCC] 7^th^ edition) were eligible for inclusion in the trial. Siewert type I and II which should be treated as esophageal cancer were excluded. The clinical stage was confirmed by imaging examinations, including thoracoabdominal enhanced computed tomography (CT) and endoscopic ultrasonography (EUS), with or without positron emission tomography CT (PET-CT). Eligible patients were aged 18–75 years and had no distant metastasis, with Karnofsky performance status (KPS) ≥ 70.

For better diagnostic accuracy of N stage and detection of distant metastasis, two radiologists with over 10 years’ specialized experience in gastrointestinal cancer reviewed the chest and abdominopelvic CT imaging, respectively. Regional lymph nodes with short-axis diameters of >5 mm with enhancement on CT images were considered metastatic. Only patients without distant metastasis as determined by the two reviewers were enrolled in the study. Laparoscopic exploration was only performed in patients with highly doubtful CT images corresponding to peritoneal dissemination (e.g., seroperitoneal or peritoneal thickening). Four to six clips were implanted around the tumor with a 1-cm margin during endoscopy before radiotherapy (RT) to aid contouring of the primary gross tumor volume (GTV).

### Neoadjuvant Chemoradiotherapy

Patients were required to be on an empty stomach for 4 hours before the CT simulation and take an oral positive contrast (300 ml) 30 minutes before the simulation to render the small intestine visible. To decrease variability in distention due to gastric filling, the patients ate a standard meal (300 ml ready-to-eat canned porridge) 15 minutes before CT scanning and before each treatment. The patients were placed in the supine position with thermoplastic immobilization during intensity-modulated RT (IMRT) with a 6-MV photon beam.

The GTV and metastatic lymph nodes (GTVnd) were delineated based on all available information (clips, EUS, CT, or PET-CT [if performed]). GTV and GTVnd with a margin of 0.5 cm in three dimensions formed the planning GTV (PGTV). The clinical target volume (CTV) consisted of GTV and GTVnd, and the elective lymphatic region as described by the Japanese Gastric Cancer Association (JGCA) (7) (**Supplementary Table S1**). The planning target volume (PTV) consisted of the CTV with a 0.5–1-cm margin in the radial direction and a 1-cm margin in the superior–inferior direction.

Simultaneous integrated boost RT (SIB-RT) was planned, which consisted of two irradiation dose levels: the patients received PTV and PGTV boost of 40.04 Gy and 45.1 Gy in 22 daily fractions, respectively. We have published the requirements for the CT simulation and dose constraints for organs at risk previously (8, 9).

The patients were required to take S-1 (80 mg/m^2^/d) 30 minutes after breakfast and after the evening meal on every RT day.

### Neoadjuvant Chemotherapy

NACT consisted of three cycles of SOX (S-1: 80, 100, 120 mg/d based on body surface area, orally daily on days 1–14; oxaliplatin, 130 mg/m^2^ intravenously on day 1, 21 days per cycle), followed by surgery.

### Surgery

patients were evaluated by CT 4 weeks after completing neoadjuvant treatment; surgery was scheduled 4–6 weeks after NACT or NACRT. D2 lymphadenectomy was strongly recommended without pancreas and spleen resection. Surgical complications were defined as any deviation from the normal postoperative course 30 days after surgery, including reoperation, wound bleeding/infection/dehiscence, anastomotic bleeding/leak, intra-abdominal bleeding/abscess, ileus.

### Adjuvant Chemotherapy

Adjuvant chemotherapy consisted of 4–6 cycles of SOX (S-1: 80, 100, 120 mg/d based on body surface area, orally daily on days 1–14; oxaliplatin, 130 mg/m^2^ intravenously on day 1, 21 days per cycle) in the NACRT group and three cycles of SOX at the same dosage in the NACT group.

### Pathological Assessment

Two pathologists reviewed the pathologic response. Tumor regression grade (TRG) was scored using the criteria of Mandard et al. (10). TRG1–2 was considered major pathological response (mPR).

### Statistical Analysis

The primary endpoint was the macroscopic and microscopic complete resection (R0 resection) rate, defined as complete resection confirmed by pathology after neoadjuvant therapy. The primary hypothesis was that the NACRT group would have a superior R0 resection rate to the NACT group. Assuming that NACRT would improve the R0 resection rate from 70% (4) to 90% as compared to NACT, a sample of 27 patients per group was planned to yield 80% power with a one-side significance level of 5% for the primary analysis. Considering a dropout rate of 20%, the study finally required 65 patients.

The secondary endpoints were pathological complete response (pCR) rate, overall survival (OS, time from randomization to death due to any cause), PFS (time from randomization to the first occurrence of locoregional failure, distant metastasis, or death from any cause in all patients), disease-free survival (DFS, time from randomization to the first occurrence of locoregional failure, distant metastasis, or death from any cause in patients with R0 resection), locoregional recurrence–free survival (LRFS, time from randomization to locoregional recurrence), distant metastasis–free survival (DMFS, time from randomization to distant metastasis) and surgical complications, toxicities, and completion rate related to protocol treatment.

**Figure 1:**
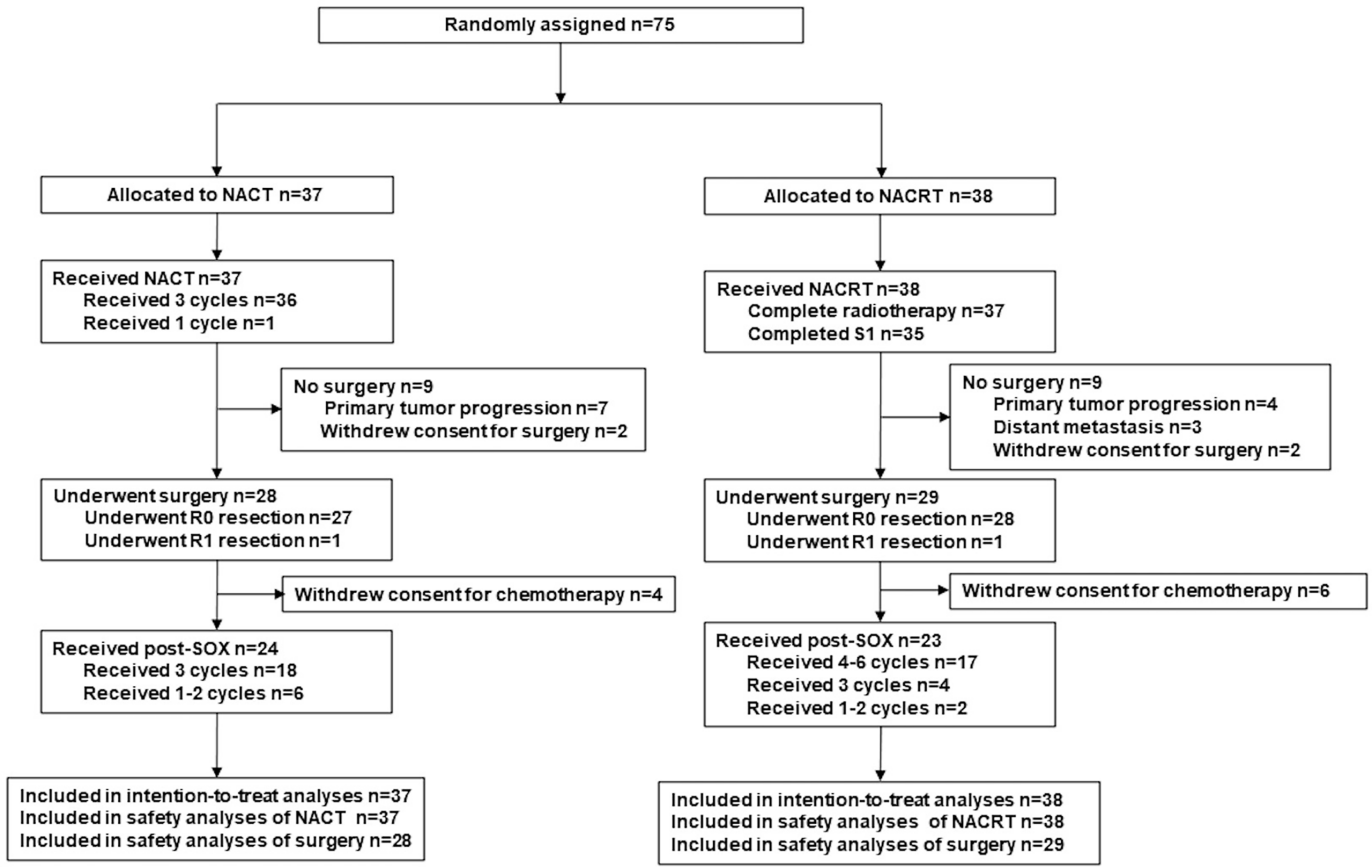
CONSORT diagram.

Efficacy analyses (OS, PFS) were evaluated in the intention-to-treat (ITT) population (defined as all enrolled patients, **Figure 1**). The data were analyzed using SPSS for Windows 21.0 (IBM SPSS Inc., Armonk, NY, USA). Differences in the proportions/rates between groups were compared using the χ^2^ test or Fisher exact test; the t-test or analysis of variance was used for continuous variables. Survival curves were calculated with the Kaplan–Meier method and compared by the log-rank test. A two-sided p-value of <0.05 was considered statistically significant.

## Results

### Patient Characteristics

Between January 2014 and October 2017, a total of 75 patients were enrolled and consecutively randomized to the NACT group (n = 37) or the NACRT group (n = 38, **Figure 1**). **Table 1** presents the clinical characteristics, which were well balanced between the two groups.

**Table 1:** Patient characteristics.

Characteristic	NACT (*n* = 37)		NACRT (*n* = 38)		*p*
	N	%	N	%	
Age (years), median (range)	58 (37-70)		57 (36-71)		0.685
Sex					
Male	30	81.1	31	81.6	0.956
Female	7	18.9	7	18.4	
WHO performance status 0 1	35 2	94.6 5.4	37 1	97.4 2.6	0.615
Location of primary tumor Upper third of stomach Middle third of stomach Lower third of stomach ≥2 sites involved	4 5 11 17	10.8 13.5 29.7 45.9	7 6 5 20	18.4 15.8 13.2 52.6	0.335
T stage (AJCC 7^th^ edition) T2/3 T4	6 31	16.2 83.8	8 30	21.1 78.9	0.591
N stage (AJCC 7^th^ edition) N1 N2 N3	6 21 10	16.2 56.8 27	12 14 12	31.6 36.8 31.6	0.168
TNM stage (AJCC 7^th^ edition) II IIIa IIIb IIIc	5 5 18 9	13.5 13.5 48.6 24.3	5 6 14 13	13.2 15.8 36.8 34.2	0.728

WHO, World Health Organization; cT, clinical T staging; cN, clinical N staging; cTNM, clinical staging.

### Neoadjuvant Therapy

All patients (n = 75) received the corresponding neoadjuvant treatment. **Table 2** shows the adverse events and toxicities (calculated using the National Cancer Institute Common Terminology Criteria for Adverse Events version 4.0) during neoadjuvant treatment. The NACRT group had a higher occurrence of anorexia (89.5% vs. 62.2%, p = 0.007), fatigue (55.3% vs. 13.5%, p < 0.001), and gastritis (36.8% vs. 5.4%, p = 0.001) of all grades than the NACT group. Neutropenia (64.9% vs. 29.0%, p = 0.003), thrombocytopenia (48.6% vs. 5.3%, p < 0.001), and elevated alanine aminotransferase or aspartate aminotransferase (13.5% vs. 0%, p = 0.025) were more common in the NACT group. However, the incidence rate of grade 3 adverse events was low and very similar between the two groups. No grade 4 or 5 toxicities were observed during neoadjuvant treatment.

**Table 2:** Overall toxicities associated with neoadjuvant treatment.

Adverse effect	NACT (*n*=37)	NACRT (*n*=38)	*P*
**All grades, No. (%)**			
Nausea	25 (67.6)	27 (71.1)	0.805
Vomiting	15 (40.5)	16 (42.1)	0.998
Anorexia	23 (62.2)	34(89.5)	0.007
Fatigue	5 (13.5)	21 (55.3)	<0.001
Gastritis	2 (5.4)	14 (36.8)	0.001
Radiation esophagitis	/	14 (36.8)	/
Hand-foot syndrome	5 (13.5)	2 (5.3)	0.262
Leukopenia	21 (56.8)	25 (65.8)	0.482
Neutropenia	24 (64.9)	11 (29.0)	0.003
Anemia	9 (24.3)	6 (15.8)	0.399
Thrombocytopenia	18 (48.6)	2(5.3)	<0.001
ALT/AST elevation	5 (13.5)	0	0.025
Grade 3, No. (%)			
Nausea	1 (2.7)	2 (5.3)	0.997
Anorexia	1 (2.7)	2 (5.3)	0.997
Fatigue	1 (2.7)	1 (2.6)	0.999
Gastritis	0	2 (5.3)	0.493
Radiation esophagitis	/	3 (7.9)	/
Leukopenia	0	3 (7.9)	0.240

### Surgical Results

Twenty-eight patients (75.7%) in the NACT group and 29 patients (76.3%) in the NACRT group proceeded to gastrectomy after neoadjuvant treatment (p = 0.999, **Figure 1**). NACRT failed to increase the R0 resection rate as compared to NACT (73.7% [28/38] vs 37.0% [27/37], p = 0.999). One patient each from the two groups underwent R1 resection, and no patient underwent R2 resection.

The reasons for not undergoing surgery in the NACT and NACRT groups, respectively, were: patient refusal (two vs. two), primary tumor progression (seven vs. four), and distant metastasis (zero vs. three).

The two groups had similar surgical time, intraoperative blood loss, and postoperative hospital stay (**Table 3**). One patient in the NACT group had postoperative complications (abdominal bleeding), as did three patients in the NACRT group (ileus, abdominal wound infection, and anastomotic bleeding each in one patient), which was not significantly different between the two groups (p = 0.611).

**Table 3:** Surgical Outcomes.

Variable	NACT	NACRT	*P*
	(*n* = 28)	(*n* = 29)	
Surgical approach, n (%)			0.491
Proximal partial gastrectomy	2 (7.1)	5 (17.2)	
Distal partial gastrectomy	12 (42.9)	12 (41.4)	
Total gastrectomy	14 (50.0)	12 (41.4)	
Surgical time, mean (SD), min	183.4 (37.1)	194.4 (49.2)	0.505
Estimated blood loss, mean (SD), ml	157.9 (170.9)	166.3 (143.1)	0.846
Postoperative hospital stay, mean (SD), d	10.5 (3.5)	10.3 (2.6)	0.913
Postoperative complication, n (%)	1 (3.6)	3 (10.3)	0.611

### Adjuvant Therapy

Adjuvant chemotherapy was performed as planned for 85.7% (24/28) and 79.3% (23/29) of patients in the NACT and NACRT arm, respectively (**Figure 1**). The other 10 patients who underwent surgery did not receive adjuvant chemotherapy because of their poor recovery from surgery. Six patients in each arm did not receive full cycles of chemotherapy due to intolerable toxicities.

### Pathological Evaluation

**Table 4** details the pathological evaluations. A median 25 lymph nodes were retrieved with NACRT, which was fewer than that with NACT (median, 37, p < 0.001). However, the two groups had a similar ratio of positive lymph nodes (median, 0.01 vs. 0, p = 0.832). pCR was achieved in 10.7% (3/28) of the NACT group and 13.8% (4/29) of the NACRT group (p = 0.999). Nevertheless, the NACRT-treated patients had better tumor response than those treated with NACT (37.9% vs 17.9%, p = 0.019).

**Table 4:** Pathological results in patients with surgery.

Variable	NACT (*n* = 28)		NACRT (*n* = 29)		*p*
	*N*	%	*N*	%	
R0 resection	27	73.0%^a^	28	73.7%^a^	0.999
D2 lymphadenectomy	26	92.9%	25	86.2%	0.611
Laparoscopic surgery	13	46.4%	17	58.6%	0.431
Tumor diameter^b^, median (range)	4.5 (2-16)		4 (1.5-14)		0.695
LNs resected, median (range)	37 (17-66)		25 (11-45)		<0.001
Positive LNs, median (range)	4.5 (1-22)		2 (1-23)		0.666
LN ratio, median (range)	0.01 (0-0.35)		0 (0-0.61)		0.832
Lauren type Intestinal Diffuse Mixed Undetermined	11 11 6 0	39.3 39.3 21.4 0	10 12 3 4	34.5 41.4 10.3 13.8	0.630
Signet ring cells Present Absent	8 20	28.6 71.4	7 22	24.1 75.9	0.704
Lymphatic/vascular invasion Present Absent	6 22	21.4 78.6	4 25	13.8 86.2	0.682
Perineural invasion Present Absent	13 15	46.4 53.6	10 19	34.5 65.5	0.358
Pathological complete response (TRG1)	3	10.7	4	13.8	0.999
Tumor response TRG1 TRG2 TRG3 TRG4 TRG5	3 2 14 9 0	10.7 7.2 50 32.1 0	4 7 17 1 0	13.8 24.1 58.6 3.5 0	0.019
ypT stage (AJCC 7^th^ edition) T0 T1 T2 T3 T4	2 2 8 5 11	7.1 7.1 28.6 17.9 39.3	4 4 7 8 6	13.8 13.8 24.1 27.6 20.7	0.490
ypN stage (AJCC 7^th^ edition) N0 N1 N2 N3	14 6 6 2	50 21.4 21.4 7.2	20 6 0 3	69.0 20.7 0 10.3	0.059
ypTNM stage (AJCC 7^th^ edition) 0 I II III	3 7 8 10	10.7 25.0 28.6 35.7	4 9 10 6	13.8 31.0 34.5 20.7	0.688

a Among intention-to-treat population.

b Maximum tumor diameter derived from surgical specimen.

LNs, lymph nodes; ypN, pathological N staging after neoadjuvant therapy; ypT, pathological T staging after neoadjuvant therapy; ypTNM, pathological staging after neoadjuvant therapy.

### Survival

The median follow-up was 59.6 months (range, 6.7–80.3 months); a total of 32 patients died (NACT group, 18; NACRT group, 14): one patient died of secondary primary tumor and 31 patients died of disease relapse.

**Figure 2:**
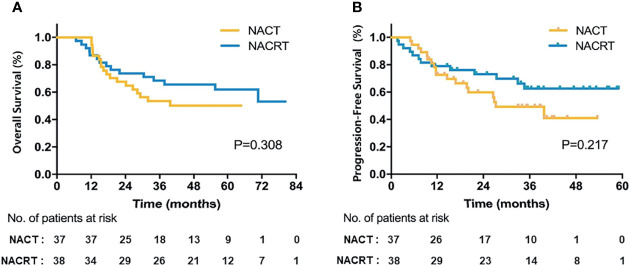
Survival of the NACT and NACRT groups in the ITT population. **(A)** OS; **(B)** PFS.

Of 75 patients in the ITT population, neither group reached the median OS (**Figure 2A**). The 3-year OS was 53.5% in the NACT group versus 68.4% in the NACRT group, and the 5-year OS was 50.1% versus 61.9%, respectively. Although the NACRT group had higher OS, there were no significant differences (p = 0.308). The median PFS of patients in the ITT population was 37.3 months with NACT and 63.4 months with NACRT (p = 0.217, **Figure 2B**).

**Figure 3:**
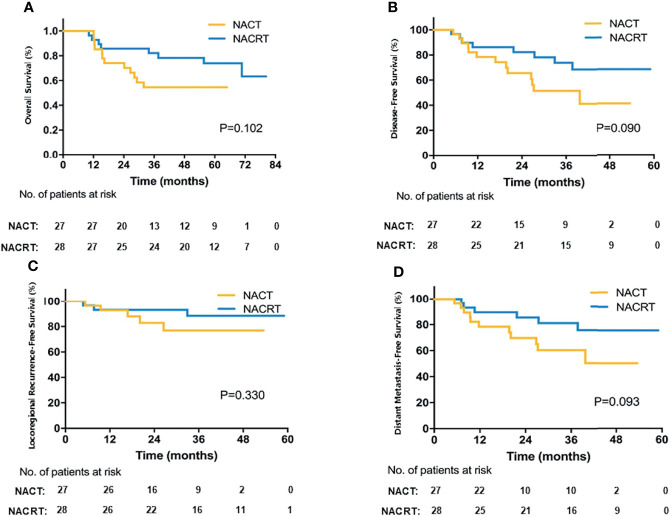
Survival of patients in the NACT and NACRT groups with R0 resection. **(A)** OS; **(B)** DFS; **(C)** LRFS; **(D)** DMFS.

Of 55 patients with R0 resection, the 3- and 5-year OS in the NACT group was 54.6% and 54.6%, respectively, and was 82.1% and 73.9%, respectively, in the NACRT group (p = 0.102, **Figure 3A**). **Figures 3B–D** and **Supplementary Table S2** show the details of the DFS, LRFS, and DMFS in the two groups after R0 resection.

Further, patients with mPR (TRG1/2) had significantly longer 5-year OS outcomes compared to TRG3 or 4 patients (85.9% vs. 62.8% vs. 29.6%, p = 0.004, **Supplementary Figure S1**). In particular, the seven patients with pCR (TRG1) benefited the most, with 100% surviving without any site of recurrence.

## Discussion

This randomized, open-label, single-center phase II trial detected no significant differences in R0 resection between the NACT and NACRT groups. The pathological outcomes suggested that patients who received NACRT had better tumor response, while the pCR rate was similar between the two groups. Both NACT and NACRT were well tolerated, with high completion rates and low postoperative complication rates.

At present, NACT and NACRT are the standard of care for patients with locally advanced resectable GC or EGJC. Numerous randomized phase III trials have proven the effect of NACT compared to surgery alone in patients with LAGC (4–6). In 2017, the FLOT regimen (docetaxel, oxaliplatin, 5-FU, leucovorin) (11, 12) was verified to confer a more notable survival benefit on patients with LAGC as a perioperative chemotherapy regimen compared to ECF. Based on these clinical results, the neoadjuvant therapeutic strategy is widely recognized. The latest published results of the CROSS trial demonstrated that, compared to surgery alone, NACRT reduced the risk of death from EC or EGJC (hazard ratio [HR]: 0.60, 95% confidence interval [CI], 0.46–0.80), with an absolute 10-year OS benefit of 13% (38% vs. 25%) (13). However, unlike NACT, which has much corresponding high-level evidence, the rationale of NACRT mostly originated from studies based on EGJC and in patients with LAGC, and requires further exploration, as only retrospective or a few single-arm phase II studies are available (14–16). Moreover, there are few published studies comparing NACT with NACRT followed by surgery in patients with LAGC, and which regimen has always been a hot discussion issue among researchers. The 2021 ASCO reported on a phase III randomized, controlled non-inferiority trial (Neo-AEGIS) that compared CROSS versus perioperative chemotherapy (modified ECF or FLOT) for locally advanced esophageal adenocarcinoma and EGJC, revealing that the 3-year estimated survival probability was 56% and 57% (HR: 1.02, 95%CI: 0.74–1.42), respectively, with no evidence that perioperative chemotherapy is unacceptably inferior to multimodal therapy (17). To our knowledge, ours is the first prospective randomized clinical trial to directly compare the efficacy and safety of the two therapeutic strategies only for patients with LAGC.

Before we conducted our study, clinicians in actual clinical practice generally believed that preoperative RT would increase the difficulty of operation and surgical complications, and there was little experience with NACRT for LAGC in China. Therefore, we performed an exploratory study of LAGC to investigate the appropriate neoadjuvant treatment modality consisting of RT technique (SIB vs. conventional), radiation dose (45 Gy vs. 50 Gy to the primary tumor), and concurrent chemotherapy regimens involving single drugs (S-1 vs. capecitabine) or double-drug agents (paclitaxel plus carboplatin, derived from the CROSS trial) (18). We found that patients could not tolerate the double-drug regimen and determined that oral S-1 (80 mg/m^2^/d) combined with 45.1/40.04 Gy SIB-RT was safe and efficacious, and was associated with a relatively lower rate of serious toxicities; 83.3% (5 of 6) of the patients with LAGC achieved R0 resection. In addition, both capecitabine and S-1 have been approved as combination treatments or monotherapies for advanced GC in East Asia, especially S-1, which is considered more efficacious for East Asian patients (19, 20). Besides, several studies have suggested that the SOX regimen is active and well-tolerated in advanced GC, and it is widely used in Southeast Asian countries (21, 22). Based on these reasons, we conducted the present study comparing NACT (SOX) with NACRT (S-1 monotherapy concurrently with RT), both followed by surgery and adjuvant chemotherapy (SOX) for LAGC. The newly published RESOLVE study confirmed the superiority of perioperative treatment with SOX compared to adjuvant chemotherapy with XELOX (capecitabine plus oxaliplatin) for patients with LAGC, which meets the requirements of clinical practice (23).

Considering that a large sample size might be required if survival outcome is considered the primary endpoint, we finally identified R0 resection as the primary endpoint in the present study, which the MAGIC trial estimated at 70% in the NACT group (4). Theoretically, concurrent chemoradiotherapy could confer more benefits for those with initially locally advanced lesions due to its tumor downstaging effect, and accordingly increase the R0 resection rate.

The results demonstrate that there were no significant differences in the NACRT and NACT groups (73.7% vs. 73.0%, p = 0.999). In the NACRT group, in addition to the two patients who refused surgery, three patients developed distant metastasis and four patients had local progression. Meanwhile, in the NACT group, except for the two patients’ refusal, all remaining seven patients had local progression, but not distant metastases, after NACT. This suggests that the intensified chemotherapy regimen played an important role in reducing the risk of distant metastasis before surgery. Accordingly, RT might be needed to increase the primary tumor response rate and further increase the probability of R0 resection. The FLOT4 study showed that the FLOT regimen was more effective and significantly increased both the pCR rate (16% vs. 6%, p = 0.02) and survival time (50 months vs. 35 months, p = 0.01) compared to ECF(X) (epirubicin, cisplatin, 5-FU/capecitabine) for patients with GC and EGJC (11, 12). Therefore, it is reasonable to combine NACRT with FLOT as an effective neoadjuvant treatment modality in LAGC, although the chemotherapy and RT sequence requires further exploration.

Here, the NACT and NACRT groups had similar pCR (TRG1) rates (10.7% vs. 13.8%, p = 0.999), which was comparable to other studies using these neoadjuvant approaches to investigate patients with LAGC (**Supplementary Table S3**) (11, 12, 14, 24–26). Except for the FLOT regimen, the pCR rate of NACT using PF (cisplatin and 5-FU) or ECF/EOX/ECX (epirubicin, cisplatin/oxaliplatin, 5-FU/capecitabine) was only 6–7% in GC and EGJC (11, 12, 25, 26). In the present study, the proportion of patients achieving pCR with SOX is relatively higher than the above results. We also found that neoadjuvant therapy compliance was high, with almost 95% of patients completing the prescribed cycles of treatment with a low incidence of grade 3 adverse events (no grade 4 adverse events occurred), which was better than that of adjuvant chemotherapy (only 59–64% of patients in both arms received the full three cycles of postoperative SOX). Therefore, considering the reduction of treatments administered postoperatively, the preoperative regimen might be intensified appropriately.

Here, the NACT-treated patients had comparable survival with that of other studies (14, 24). Although the NACRT group had a longer median OS and PFS, the p-values had no statistical significance compared to the NACT group. However, this result should be interpreted with caution, and may have arisen by chance, as our study was not powered for survival and the samples were relatively small. The mPR patients did not develop locoregional recurrence and had significantly longer 5-year OS (85.9%), DFS (93.8%), and DMFS (93.8%) compared with TRG3–4 patients, irrespective of preoperative therapy. We believe that the TRG might be a factor that affected the prognosis, and the increased survival outcomes were partly related to the better tumor response (p = 0.019) in the NACRT group. Martin-Romano et al. reported that the high 5-year OS rate of >70% in patients with GC was associated with favorable tumor regression (15). However, the strong correlation in GC between tumor response after neoadjuvant therapy and long-term survival benefit requires confirmation in the future.

This study has some limitations. First, it was a single-center, small-sample study. Second, we did not perform routine laparoscopy or PET-CT for staging diagnosis, as PET-CT has not been proven to be highly effective in M1 staging for GC, while laparoscopic exploration is invasive and may delay the time to start treatment; both might result in more resistance against enrollment. Therefore, two experienced radiologists specialized in gastrointestinal cancer were assigned to perform each patient’s staging evaluation to ensure better diagnostic accuracy. If peritoneal metastasis was highly suspected, laparoscopic exploration was performed, and the patient was excluded before enrollment if the lesions were confirmed.

## Conclusions

In conclusion, S-1-based NACRT did not improve the R0 resection rate despite better tumor regression and a similar safety profile to NACT for LAGC. Long-term follow-up revealed a trend of improved survival outcomes in the NACRT group, which requires confirmation in a prospective, phase III trial.

## Data Availability Statement

The original contributions presented in the study are included in the article/**Supplementary Material**. Further inquiries can be directed to the corresponding authors.

## Ethics Statement

The studies involving human participants were reviewed and approved by National Cancer Center ethics committee. The patients/participants provided their written informed consent to participate in this study.

## Author Contributions

Conception and design of the study: XW, DZ, LY and JiJ, Study conduct: XW, DZ, LY, YC, HZ, L-MJ, JuJ, YT, NL, W-YL, L-ZD, S-MZ, L-YX, Y-TT, XC, C-GG, X-FB, Y-MS, S-LW, Y-WS, Y-PL, HF, Y-XL, and JiJ, Data collection: XW, NL and W-YL. Data analysis: XW and J-SR, Drafting the manuscript: XW, Y-XL and JiJ, Revising the manuscript content: all authors, Final approval of the version to be submitted: all authors.

## Funding

This work was supported by grants from the Beijing Marathon of Hope Foundation (LC2018L03), Shenzhen Key Medical Discipline Construction Fund (SZXK013), and Sanming Project of Medicine in Shenzhen (SZSM201612063), and the Foundation of Clinical Society of Clinical Oncology (Y-SY201901-0004).

## Conflict of Interest

The authors declare that the research was conducted in the absence of any commercial or financial relationships that could be construed as a potential conflict of interest.

## Publisher’s Note

All claims expressed in this article are solely those of the authors and do not necessarily represent those of their affiliated organizations, or those of the publisher, the editors and the reviewers. Any product that may be evaluated in this article, or claim that may be made by its manufacturer, is not guaranteed or endorsed by the publisher.
