# Supplementary material for: Preoperative Concurrent Chemoradiotherapy Versus Neoadjuvant Chemotherapy for Locally Advanced Gastric Cancer: Phase II Randomized Study
Source: Front Oncol. 2022 Apr 29;12:870741. doi: 10.3389/fonc.2022.870741 (PMC9104815; doi:10.3389/fonc.2022.870741)

Supplement Materia S1

Table S1. CTV for elective nodal regions based on the primary gastric tumor location according to JGCA guidelines

Tumor location	CTV for elective nodal regions
Upper 1/3	110, 1-3, 4[a], 7-11, 16a
Middle 1/3	1-3, 4*, 5-9, 11p, 12, 16a
Lower 1/3	3, 4*, 5-9, 11p, 12, 16a

110, Paraesophageal lymph nodes (LNs) in the lower thorax; 1, right paracardial LNs; 2, left paracardial LNs; 3, LNs along the lesser curvature; 4, LNs along the greater curvature; 5, suprapyloric LNs; 6, infrapyloric LNs; 7, LNs along the left gastric artery; 8, LNs along the common hepatic artery; 9, LNs around the celiac artery; 10, LNs at the splenic hilum; 11, LNs along the splenic artery (11p, LNs along the proximal splenic artery); 12, LNs in the hepatoduodenal ligament; 16a, LNs around the abdominal aorta (above the level of the inferior border of the left renal vein).

[a]No. 4 was included in the CTV only when the tumor involved the greater curvature.

Table S2. Survival of patients with R0 resection

Variable	NACT (n =27)	NACRT (n =28)	P
OS			0.102
2-year	74.1%	85.7%	
3-year	54.6%	82.1%	
5-year	54.6%	73.9%	
DFS			0.090
2-year	65.5%	82.3%	
3-year	51.5%	73.8%	
5-year	41.2%	68.6%	
LRFS			0.330
2-year	82.8%	93.1%	
3-year	76.9%	88.5%	
5-year	76.9%	88.5%	
DMFS			0.093
2-year	69.7%	85.5%	
3-year	60.4%	81.2%	
5-year	50.3%	75.8%	

Table S3. Results of NACRT or NACT for patients with LAGC

Author (year)	Study design	Patients (n)	cTNM (%)	ypTNM (%)	NACT or NACRT	R0/pCR/pPR (%)	Survival
Kim, et al (2015) [14]	RS	29	NM	T4, 93; N+, 79	FP/SP +45Gy	59/11/28	MST 21m, 1-y OS 72%
Trip, et al (2014) [24]	PS	25	T4, 36; N+, 92	T4, 0; N+, 56	PC+45Gy	72/16/24	MST 15m
Schuhmacher, et al (2010) [25]	RCT	NACT: 72	T3-4, 100 N+, 94.4	T3-4, 34; N+, 61	FP	85/6/30	MST 65m, 2y OS 73%
Al-Batran et al (2016, 2019) [11,12]	RCT	ECF(X); 137 FLOT: 128	T3-4, 82; N+, 78	ECF(X): T3-4, 73; N+, 47; FLOT: T3-4, 56; N+, 41;	ECF(X) vs FLOT	ECF(X): 74/6/17 FLOT: 85/16/21	ECF(X): 3y OS 48%, MST 35m; FLOT: 3y OS 57%; MST 50m
Cats et al (2018) [26]	RCT	NACT: 393[a]	NM	T3-4, 45; N+, 52	EC(O)X	80/7/NM	5y OS 42%; MST 43m
Our study	RCT	NACT: 37; NACRT: 38	T4, 81; N+, 100	NACT: T3-4, 57; N+, 50; NACRT: T3-4, 44; N+, 31;	NACT: SOX; NACRT: S-1+45.1/40.04Gy	NACT: 71/11/7 NACRT: 72/14/24	NACT: 5y OS 50% NACRT: 5y OS 62% MST: both not reached

cTNM, Clinical stage grouping; ECF(X), epirubicin, cisplatin, and 5-FU/capecitabine; FLOT, docetaxel, oxaliplatin, fluorouracil, and leucovorin; FP, 5-FU and cisplatin; MST, median survival time; NACRT, neoadjuvant chemoradiotherapy; NACT, neoadjuvant chemotherapy; NM, not mentioned; PC, paclitaxel and carboplatin; RCT, randomized controlled trial; RS, retrospective study; PS, prospective study; pCR, complete pathological response (corresponding to Becker Ia or Mandard TRG1); pPR, subtotal histopathological regression (corresponding to Becker Ib or Mandard TRG2); SP, S-1 and cisplatin; ypTNM, pathological staging following neoadjuvant therapy.

[a]In perioperative chemotherapy arm without postoperative chemoradiotherapy.

Figure S1. Survival according to TRG in patients with R0 resection. (a) OS; (b) DFS; (c) LRFS; (d) DMFS.

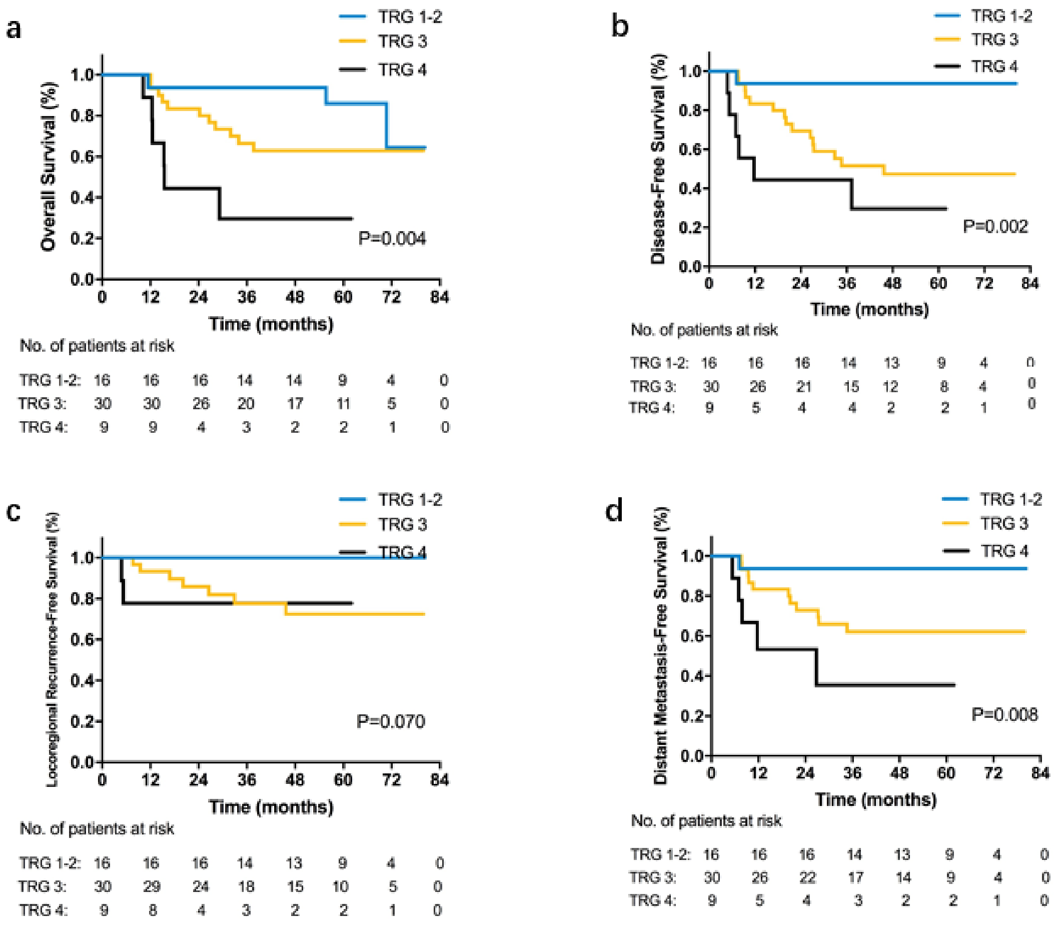

Supplement: Supplementary file 1 [file DataSheet_1.pdf]
